# Acute Cheilitis Associated with Oseltamivir after Influenzae A Infection

**DOI:** 10.1155/2023/8618245

**Published:** 2023-12-06

**Authors:** Koji Yokoyama, Mitsukazu Mamada

**Affiliations:** Department of Pediatrics, Japanese Red Cross Wakayama Medical Center, Wakayama, Japan

## Abstract

Oseltamivir is a neuraminidase inhibitor used to treat acute influenza A or B in adult and pediatric patients. Adverse reactions are usually mild. Here, we report novel side effects associated with oseltamivir. The patient was an 11-year-old girl who developed lower lip cheilitis and stomatitis, after taking oseltamivir. Her symptoms and signs resolved within 36 h of oseltamivir discontinuation. She has clinically fully recovered and has remained well.

## 1. Case Presentation

**Figure 1:**
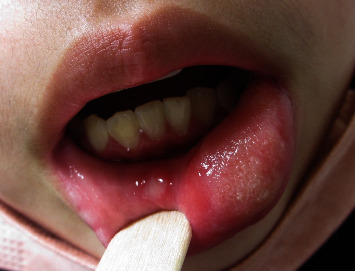
Image of her white swollen lip and an ulcer on the labia.

An 11-year-old girl was admitted to our hospital with a 1 day history of lower lip cheilitis and stomatitis. No other part of her body or face had any swelling. She had no history of food, drug allergies, and inhalant abuse. It was the first time this had happened. She had no abdominal pain, no history of allergies or eczema, and no family history of angioedema. She had started taking oseltamivir capsules (75 mg twice daily) 2 days before the swelling began, having visited her primary care doctor with a bout of seasonal influenza with fever, cough, and arthralgia. These symptoms eased gradually while taking oseltamivir. She also took an expectorant (dextromethorphan) and an anti-inflammatory drug (tiaramide hydrochloride) for 2 days after visiting her doctor. She has taken an expectorant and an anti-inflammatory drug many times before without any problems. She was not taking any other prescription medications, including antiepileptic drugs or antibiotics. She was febrile (38.0°C), had a white swollen lip, an ulcer on the labia, and bilateral cervical lymphadenopathy (Figure 1). There were no rash or meningeal signs. She had no vulvar ulcers, erythema nodosum, folliculitis-like rash, or ophthalmological abnormalities. The remainder of the physical examination was unremarkable. Laboratory investigations revealed leukopenia (WBC, 2.03 × 10^9^/L); neutrophil granulocytes, 64.6%; elevated C-reactive protein (CRP, 1.48 mg/mL), and no vitamin deficiencies. No significant results were found in comprehensive respiratory virus testing or antibody titer studies. Oseltamivir was discontinued, and she was treated with an expectorant and anti-inflammatory drug. Her symptoms and signs resolved within 36 h of oseltamivir discontinuation. She has been followed up for over a year, no recurrence of symptoms has been observed. The possible reaction of oseltamivir was reported to Chugai Pharmaceutical Co., Ltd. The guardian of the patient must provide written consent for the data to be published.

## 2. Discussion

The course of this patient raises two important clinical points. First, patients taking oseltamivir can suffer from lower lip cheilitis and stomatitis. Second, these two conditions can occur simultaneously in pediatric patients. Oseltamivir is a neuraminidase inhibitor used to treat acute influenza A or B in adult and pediatric patients. Adverse reactions are usually mild. Oseltamivir-induced oral reactions have been reported, particularly tongue swelling, which has been observed and reported in postmarketing surveillance [1, 2]. Glossal swelling associated with oseltamivir is likely to be an idiosyncratic drug reaction, likely caused by an immune-mediated reaction to metabolites or by direct cytotoxicity of the drug or its metabolites [1]. An IgE-mediated allergic reaction is also possible. However, the patient's IgE levels were normal, and she had no history of allergic disease. Drug-related cheilitis is one type of reversible cheilitis that can be caused by retinoids and antiviral drugs [3]. The prevalence of influenza and the dispensation of antiflu agents are higher in children than in adults [4]. Idiosyncratic reactions can occur even in children (indeed, age may be a factor), and these reactions can be unpredictable and are not fully understood [5]. The pathogenesis of oseltamivir-induced cheilitis and stomatitis is not well understood, and future studies are needed to ascertain how oseltamivir causes oral reactions, whether predisposing factors exist and the actual incidence of this condition. More cases need to be recorded if we are to better understand the pathology of drug-associated oral reactions.

Physicians should pay attention to the various side effects of drugs.
